# Hospital-acquired *Clostridium difficile-associated *disease in the intensive care unit setting: epidemiology, clinical course and outcome

**DOI:** 10.1186/1471-2334-7-42

**Published:** 2007-05-21

**Authors:** Alexandre R Marra, Michael B Edmond, Richard P Wenzel, Gonzalo ML Bearman

**Affiliations:** 1 Department of Infectious Diseases, Universidade Federal de São Paulo, São Paulo, Brazil; 2 Department of Internal Medicine, Medical College of Virginia Campus, Virginia Commonwealth University, Richmond, Virginia, USA

## Abstract

**Background:**

*Clostridium difficile*-associated disease (CDAD) is a serious nosocomial infection, however few studies have assessed CDAD outcome in the intensive care unit (ICU). We evaluated the epidemiology, clinical course and outcome of hospital-acquired CDAD in the critical care setting.

**Methods:**

We performed a historical cohort study on 58 adults with a positive *C. difficile *cytotoxin assay result occurring in intensive care units.

**Results:**

Sixty-two percent of patients had concurrent infections, 50% of which were bloodstream infections. The most frequently prescribed antimicrobials prior to CDAD were anti-anaerobic agents (60.3%). Septic shock occurred in 32.8% of CDAD patients. The in-hospital mortality was 27.6%. Univariate analysis revealed that SOFA score, at least one organ failure and age were predictors of mortality. Charlson score ≥3, gender, concurrent infection, and number of days with diarrhea before a positive *C. difficile *toxin assay were not significant predictors of mortality on univariate analysis. Independent predictors for death were SOFA score at infection onset (per 1-point increment, OR 1.40; CI95 1.13–1.75) and age (per 1-year increment, OR 1.10; CI95 1.02–1.19).

**Conclusion:**

In ICU patients with CDAD, advanced age and increased severity of illness at the onset of infection, as measured by the SOFA score, are independent predictors of death.

## Background

*Clostridium difficile *is a major cause of antibiotic associated diarrhea and colitis [1]. The incidence of infection with this organism is increasing in hospitals worldwide, consequent to the widespread use of broad-spectrum antibiotics [2].

Hospital-acquired *C. difficile *disease is associated with not only antimicrobial use, but also advanced age, laxative use, proton pump inhibitors, antineoplastic chemotherapeutic agent use, renal insufficiency, and gastrointestinal surgery or procedures [3,4].

Over the past several years, numerous reports have been published regarding CDAD in immunocompromised patients (bone marrow transplantation, solid organ transplantation, HIV infected patients and those with neutropenia), [5-9] as well as hospitalized patients in acute care medical wards [10].

The clinical and financial impact of nosocomial CDAD, as measured by the attributable cost and length of stay, is significant [11]. However few studies have assessed CDAD outcome in the intensive care unit (ICU) setting [12].

The purpose of this study was to evaluate the epidemiology, clinical course and outcome of hospital-acquired CDAD in the ICU setting.

## Methods

### Setting

The Virginia Commonwealth University Medical Center (VCUMC) is an 820-bed tertiary care facility in Richmond, Virginia. The hospital houses 9 intensive care units (ICUs), including pediatric ICUs and a burn unit. Approximately 30,000 patients are admitted annually. The study was approved by the VCUMC Institutional Review Board (IRB).

### Study design

All ICU patients with a positive *C. difficile *toxin at VCU Medical Center from January 2002 to August 2005 were identified retrospectively using the electronic medical microbiology record. This was followed by a retrospective chart review. Each patient was only included once at the time of the first *C. difficile *result. Patients older than 18 years of age were included in the analysis. At study entry, we recorded patient age, gender, location (medical ICU or surgical ICU), predisposing clinical conditions on admission, and colonoscopy or surgical procedures for CDAD. Data on nosocomial infections were collected for 14 days following the date of the first positive *C. difficile *toxin result. Additionally, we collected data on the duration of diarrhea before and after diagnosis and treatment of CDAD. Antimicrobial exposure preceding the diagnosis of CDAD and the interruption of antimicrobial treatment due to CDAD were also collected. Colonization status with methicillin-resistant *S. aureus *(MRSA) and vancomycin-resistant enterococci (VRE) were identified by clinical cultures during the time of study. Adverse outcomes (organ failure and mortality) that occurred during the hospital stay were recorded. The clinical condition of each patient was classified daily according to systemic inflammatory response syndrome (SIRS) criteria [SIRS, sepsis, severe sepsis or septic shock] and SOFA scores from two days prior to the first positive stool *C. difficile*-toxin assay through 14 days afterwards [13,14]. The Sequential Organ Failure Assessment (SOFA) score, assesses the incidence and severity of organ dysfunction in critically ill patients [14]. The severity of underlying disease preceding CDAD was classified using the Charlson weighted comorbidity index [15].

### Definitions

We defined a CDAD case as a patient with a positive stool *C. difficile *cytotoxin assay result and diarrhea. Diarrhea was defined as a change in bowel habit with ≥3 unformed bowel movements per day for ≥2 days [10]. We defined treatment failure as recurrence of diarrhea plus an additional stool specimen positive for *C. difficile *toxin or a physician order for a second course of treatment for *C. difficile*. Concurrent infection detected during CDAD was defined according to Centers for Disease Control and Prevention (CDC) criteria [16]. P revious antibiotic treatment was defined as an antibiotic prescribed for at least 48 hours in the 60 days prior to CDAD. The SOFA score was used to assess disease severity [14]. Systemic Inflammatory Response Syndrome (SIRS) was defined as two or more of the following: (1) temperature >38°C or <36°C, (2) heart rate >90 beats per minute, (3) respiratory rate >20 breaths per minute or a PaCO_2 _<32 mmHg, or (4) white blood cell count >12 × 10^9^/L or <4 × 10^9^/L or the presence of more than 10% immature neutrophils.

The clinical condition of each patient with CDAD was classified daily as SIRS, sepsis, severe sepsis or septic shock using criteria previously published by the American College of Chest Physicians/Society of Critical Care Medicine (ACCP/SCCM) [13]. Sepsis was defined as SIRS associated with *C. difficile*-associated diarrhea. Sepsis associated with organ dysfunction, hypotension or systemic manifestations of hypoperfusion constituted severe sepsis. Septic shock was defined as sepsis associated with hypotension unresponsive to intravenous fluid challenge or the need for a vasopressor agent. The presence of organ system failure was assessed using the criteria described by Fagon [17]. Nosocomial infection was defined as an infection that occurred >48 hours after hospital admission, an infection that occurred <48 hours after admission to the hospital in patients that had been hospitalized in the 3 weeks prior to the admission, or an infection that occurred <48 hours after admission to the hospital in patients that had been transferred from another hospital or nursing home [16]. Treatment for CDAD was defined as the receipt of metronidazole at a dosage of 250 mg 4 times per day or 500 mg 3 times per day for 10–14 days, or oral vancomycin 125 mg 4 times per day for the same duration.

### Statistical analysis

Continuous variables were compared using the Student *t *test for normally distributed variables and the Mann-Whitney U test for non-normally distributed variables. Differences in proportions were compared using a Chi-square test or Fisher exact test when appropriate. Alpha was set at 0.05 and all tests of significance were two-tailed. Variables significant for predicting mortality in univariate analysis were entered into a logistic regression model. When collinearity was identified between two variables in a correlation matrix, the one with the greatest clinical relevance associated with mortality was included in the final multivariate analysis. The Hosmer and Lemeshow goodness-of-fit statistic was used to assess adequacy of the model fit. Odds ratios with 95% confidence intervals were calculated for independent variables associated with mortality related to CDAD. All statistical analyses were done using the Statistical Package for the Social Sciences software (SPSS version 11.0, Chicago, IL, USA).

## Results

Study population and patient characteristics. From 613 hospitalized patients with CDAD, a total of 70 patients were identified at VCUMC ICUs during the study period. Sixty-three patients (90%) were adults and 7 were pediatric patients (10%). Of the adult patients, 5 (7.9%) had incomplete medical records. The remaining 58 adult ICU patients with CDAD were included in the analysis.

The median age was 55.5 years with an inter-quartile range (IQR) from 43 to 64 years. Twenty-two patients (37.9%) were over 60 years of age. The majority of patients were from surgical ICUs (63.8%). The most frequent admission diagnoses were trauma (22.4%), cardiovascular disease (20.7%), gastrointestinal diseases (17.2%) and solid and haematologic malignancies (12.1%). The mean interval between hospitalization and CDAD infection was 16.8 ± 18.5 days. The median duration of hospitalization was 32.0 days (IQR = 16.7–68.2). The mean duration of diarrhea before CDAD diagnosis was 6.5 ± 7.0 days. The majority of patients (93.1%) were receiving antimicrobial therapy before CDAD (Table 1). The most frequently prescribed antimicrobials prior to CDAD were anti-anaerobic agents considering piperacillin-tazobactam, metronidazole, carbapenems, or clindamycin (60.3%), quinolones (43.1%), and intravenous vancomycin (43.1%). Other prescribed antimicrobials were also cephalosporins (36.2%) and aminoglycosides (15.6%).

**Table 1:** Characteristics of CDAD in ICU patients.

**VARIABLES**	**N**	**%**
**Age, years**		
Median (IQR)	55.5 (43.0–64.0)	-
≤65	47	81.0
>65	11	19.0
**Male gender**	35	60.3
**Charlson index, median (range)**	2 (0–8)	-
**Intensive care unit**		
Medical	21	36.2
Surgical	37	63.8
**Diagnosis on admission**		
Trauma	13	22.4
Cardiovascular disease	12	20.7
Gastrointestinal disease	10	17.2
Neoplasm	7	12.1
Respiratory disease	6	10.3
Neurologic disease	4	6.9
Other	6	10.3
**Interval between hospitalization and CDAD, mean days ± SD**	16.8 ± 18.5	-
**Length of hospital stay: median days (IQR)**	32.0 (16.7–68.2)	-
**Days with diarrhea before positive *C. difficile *toxin assay, mean ± SD (range)**	6.5 ± 7.0 (0–31)	-
**Received antimicrobial therapy prior to CDAD**	54	93.1
**Concomitant infection**	36	62.1
**Initial antibiotic treatment course**		
Metronidazole	57	98.3
None	1	1.7
Second antibiotic treatment course		
Not required	53	91.4
Metronidazole	2	3.4
Vancomycin	1	1.7
Metronidazole and vancomycin	2	3.4
**Continued to receive antimicrobial therapy after CDAD**	49	84.5
Concurrent MRSA or VRE colonization	18	31.0
In-hospital mortality	16	27.6

Sixty-two percent of patients (36/58) had a concurrent infection; of these 50% (18/36) were bloodstream infections (BSIs), a sizable fraction of which (39.9%, 11/18) were polymicrobial. The most frequent concurrent BSI pathogens BSI were gram-negative bacteria (35.0%), following by coagulase-negative staphylococci (20.0%), and *Candida albicans *(20.0%). Other isolated pathogens *Enterococcus *spp (15.0%), *Streptococcus viridans *(5.0%) and *Candida glabrata *(5.0%). No vancomycin-resistant enterococci were detected. In approximately thirty-nine percent of BSI patients (7/18), a 24 hours delay in the initiation of appropriate antimicrobial therapy was observed.

Nearly all patients (98.3%) with CDAD received metronidazole as the initial antimicrobial treatment. Five patients failed the first course of antimicrobial therapy (8.6%); of these, two were treated with a second course of metronidazole, one was treated with vancomycin and two patients received both drugs (Table 1). The majority of patients (84.5%) with CDAD received concurrent antimicrobial therapy; in 13 (22.4%) of these patients there was no evidence of another infection. Two patients (3.4%) underwent colonoscopy for CDAD, but no patients required operative management. Almost one-third of CDAD patients were colonized with MRSA or VRE (31.0%).

### Clinical course

Septic shock occurred in 32.8% (19/58) of CDAD patients. The maximal SIR (severe sepsis, septic shock or death) occurred in 43.1% (25/58) during the 14 days following the first positive *C. difficile *toxin assay. No significant difference was observed in maximal SIR score between patients with CDAD only and those with concurrent nosocomial infection (31.8% vs. 50.0%, p = 0.17). A statistically significant difference in SOFA scores was observed between the survivors and non-survivors. The 14-day mortality was 17.2% (10/58) and the in-hospital mortality was 27.6% (16/58).

Univariate analysis revealed that SOFA score, septic shock, at least one organ failure (respiratory, cardiovascular, renal, hematologic and hepatic) and age were significant predictors of mortality (table 2). Charlson score ≥3, gender, other concurrent nosocomial infections, and days from start of diarrhea to start of treatment for CDAD were not significant predictors of mortality on univariate analysis. Variables significant for predicting mortality in univariate analysis (age, SOFA score and at least one organ failure) were entered into a logistic regression model (p < 0.05). Using this model, the following variables were independent predictors for death (table 3): SOFA score at CDAD infection onset (per 1-point increment; OR 1.40, CI95 1.13–1.75) and age (per year; OR 1.10, CI95 1.02–1.19).

**Table 2:** Univariate analysis of risk factors for in-hospital mortality in CDAD patients.

	**Died (n = 16)**		**Recovered (n = 42)**		***P***
	
**VARIABLES**	N	%	N	%	
**Age, median years (IQR)**	61.0 (56.0–69.0)	-	53.0 (36.7–63.0)	-	0.015
**Male**	7	43.8	28	66.7	0.11
**Charlson ≥3**	5	31.3	12	28.6	1.0
**SOFA score at infection onset, median (IQR)**	9.5 (6.0–14.5)	-	4.0 (2.0–6.0)	-	<0.001
**Other infections concurrently**	11	68.8	25	59.5	0.52
**Days of diarrhea prior to positive *C. difficile *toxin assay, median (IQR)**	7.5 (2.0–14.2)	-	3.0 (2.0–7.0)	-	0.32
**Organ failure (any)**	16	100.0	28	66.7	0.01
**Septic shock**	12	75.0	7	16.7	<0.001
**Respiratory failure**	14	87.5	22	52.4	0.014
**Renal failure**	11	68.8	6	14.3	<0.001
**Hematologic failure**	3	18.8	3	7.1	0.33
**Hepatic failure**	5	31.3	3	7.1	0.03

IQR, Inter-quartile range

**Table 3:** Multivariate analysis of factors independently associated with death in ICU patients with CDAD.

**VARIABLES**	**OR**	**95%CI**	***P***
**Age, years (per 1-year increment)**	1.10	1.02–1.19	0.011
**SOFA score at infection onset (per 1-point increment)**	1.40	1.13–1.75	0.002
Organ failure (any)*	-	-	0.998

#Hosmer and Lemeshow goodness-of-fit statistic = 7.1 (8 DF) (p = 0.53)

OR, odds ratio

CI, confidence interval

*Organ failure occurred in 100% who died from CDAD, as such, there is no odds ratio and confidence interval

## Discussion

To better understand the epidemiology of CDAD infection in critically ill patients, we investigated its clinical course and outcome in the intensive care units of a tertiary care, academic medical center. Of the 58 ICU patients with *C. difficile *colitis in our cohort study, a significant proportion of these patients had concurrent infection (62.1%). The majority of the pathogens associated with nosocomial infections in our study cohort were gastrointestinal flora. This finding coupled with the relatively high proportion of bloodstream infections that were polymicrobial suggest that the inflammation and disruption of the colonic mucosa by CDAD provided a source of entry for organisms into the bloodstream. Of note, 60% of these patients had recently been treated with antimicrobials with significant activity against anaerobes, suggesting an important protective role that anaerobes may play in the intestinal flora. Similarly, disruption of the anaerobic gut flora has been shown to be a risk factor for VRE colonization [18], an epidemiologic finding that has been experimentally validated in an animal model [19].

As more than a half of our *C. difficile *patients were co-infected, findings predictably showed that a high proportion of ICU CDAD patients had a maximal SIR (severe sepsis, septic shock or death) during 14 days of follow-up. However, no statistically difference was observed in maximal SIR between patients with CDAD only those with CDAD and another infection (p = 0.17). One potential explanation for this observation may have been the delay in the introduction of specific antimicrobial therapy (metronidazole or oral vancomycin) for patients with CDAD. In our study, ICU patients were symptomatic with diarrhea on average for 6.5 days prior to microbiologic confirmation of the CDAD diagnosis and initiation of therapy. In our study cohort, no patients required surgical procedures as a result of CDAD. This is similar to another report in which 2.5% of patients required emergency colectomy [20].

Some studies have shown that treatment of CDAD with metronidazole may lead to poor outcomes [21,22]. In our study it was not our purpose to determine the efficacy of metronidazole since we had multiple confounders such as a high rate of co-infection, and some patients died before completing metronidazole treatment. Almost one-fifth of our patients received antibiotics in the absence of evidence to suggest a concurrent infection. This observation is particularly important as prior reports suggest that unnecessary antibiotic use makes treatment of CDAD more difficult and potentially less efficacious [2,3].

The crude mortality rate associated with *C. difficile *diarrhea in our ICU population was high (27.6%). Similarly, a prospective study at 12 Quebec hospitals of 1,073 patients with CDAD found a 30-day crude mortality rate of 24.8% independent of being ICU or non-ICU patients [23]. In this study, Loo et al. found that the age-specific incidence of CDAD increased markedly after the age of 50 years and the attributable mortality rate increased after the age of 60 years [23]. Pepin et al. have also found that advanced age (≥65 years) is a risk factor for recurrence of CDAD following treatment with metronidazole. By multivariate analysis, we demonstrated that for ICU patients with CDAD, advanced age and a high SOFA score were independent predictors of mortality. Prior studies have identified advanced age as a risk factor for CDAD [11,24]. To our knowledge, no other studies have reported SOFA score as a predictor for mortality in ICU patients with CDAD.

The limitations of our study should be acknowledged. First, we performed a retrospective cohort study; second, we only evaluated patients during hospitalization. Thus, we cannot comment on long term follow-up, including relapse of diarrhea and mortality. Third, these findings were specific for an ICU population in a tertiary care medical center and thus may not be generalizable to a non-ICU population. Fourth, as this study lacked controls, we did not investigate risk factors for the development of CDAD in our ICU population. Lastly, the study had a small sample size thereby potentially resulting in a type II error at the time of univariate and multivariate analysis.

## Conclusion

CDAD is a serious nosocomial infection in ICU patients. It is associated with a high mortality and a high rate of nosocomial co-infection. As such, the management of these infections remains challenging. In ICU patients with CDAD, advanced age and increased severity of illness at the onset of infection, as measured by the SOFA score, are independent predictors of death.

## Key messages

CDAD is not an infrequent infection with an usually late diagnosis and therapy, and high potential comorbidity and mortality. Almost one-fifth of our patients received antibiotics in the absence of evidence to suggest a concurrent infection. Antibiotics are used in a indiscriminate way in ICU. This observation is particularly important as prior reports suggest that unnecessary antibiotic use makes treatment of CDAD more difficult and potentially less efficacious.

## Competing interests

The author(s) declare that they have no competing interests.

## Authors' contributions

ARM participated in the design of the study, collected the data and performed the statistical analysis. GMLB participated in the design of the study and performed the statistical analysis. RPW participated in the design of the study and coordination. MBE conceived of the study, and participated in its design and coordination. All authors read and approved the final manuscript.

## Pre-publication history

The pre-publication history for this paper can be accessed here:


